# FiNGS: high quality somatic mutations using filters for next generation sequencing

**DOI:** 10.1186/s12859-021-03995-y

**Published:** 2021-02-18

**Authors:** Christopher Paul Wardell, Cody Ashby, Michael Anton Bauer

**Affiliations:** Department of Biomedical Informatics, University of Arkansas for Medical Sciences, 4301 W Markham St, Little Rock, AR 72205 USA

**Keywords:** Sequence analysis, Next generation sequencing, Cancer, Genomics, Sequencing, DNA, Mutations, Snvs, Filtering, Quality control

## Abstract

**Background:**

Somatic variant callers are used to find mutations in sequencing data from cancer samples. They are very sensitive and have high recall, but also may produce low precision data with a large proportion of false positives. Further ad hoc filtering is commonly performed after variant calling and before further analysis. Improving the filtering of somatic variants in a reproducible way represents an unmet need. We have developed Filters for Next Generation Sequencing (FiNGS), software written specifically to address these filtering issues.

**Results:**

Developed and tested using publicly available sequencing data sets, we demonstrate that FiNGS reliably improves upon the precision of default variant caller outputs and performs better than other tools designed for the same task.

**Conclusions:**

FiNGS provides researchers with a tool to reproducibly filter somatic variants that is simple to both deploy and use, with filters and thresholds that are fully configurable by the user. It ingests and emits standard variant call format (VCF) files and will slot into existing sequencing pipelines. It allows users to develop and implement their own filtering strategies and simple sharing of these with others.

## Background

Somatic variant callers find mutations in cancer samples by comparing sequencing data from matched tumor-normal sample pairs and they output lists of the differences they detect. These differences are a mixture of true somatic mutations and false positives. Confounding factors such as the purity of the samples, the sub-clonal heterogeneity of cancer samples, somatic copy number aberrations, artifacts introduced by sequencing chemistry, the alignment algorithm and the incomplete and repetitive nature of reference genomes all lead to somatic variant calls that are rich in false positives. Furthermore, somatic variant callers are optimized to be fast, as in many cases they must traverse the entire genome and the sequencing data could potentially be thousands of reads deep.

It is common practice for sequencing studies to attempt to ameliorate these effects using a variety of filtering techniques, including taking the intersect of results from multiple variant callers and employing some form of post-calling filtering.

This ad-hoc filtering varies greatly between laboratories, leading to issues in data handling practices and reproducibility. Even if the filtering heuristics used are fully and accurately reported in the methods, other laboratories must produce their own code to reproduce these filters.

Besides in-house scripts, there are limited options available for filtering variants, each with their own compromises. VCFtools [1] can only filter variants based on data encoded in the VCF file itself, which is by design a sparse summary of the data. FPfilter is a script developed as part of the VarScan2 variant caller [2], but is intended for use with that variant caller and only considers the tumor binary alignment map (BAM) file. As of the latest build (version 4) of the Genome Analysis Tool Kit (GATK) [3], the filtering of MuTect calls is broken out into a separate step called FilterMutectCalls. While this does output a standard VCF file, it only works with VCFs produced using the MuTect variant caller and requires users to fully commit to the GATK software ecosystem.

Attempts have been made to standardize filtering methodology between laboratories, with recommendations produced by the International Cancer Genome Consortium (ICGC) [4]. Despite this, there has not yet been a filtering tool released that implements this methodology. Therefore, we have developed Filters for Next Generation Sequencing (FiNGS), software written specifically to address these issues and also provide an implementation of the ICGC filtering standards.

FiNGS is robust, easy to both use and integrate into existing software pipelines. Further, it is flexible, so that users may pick and choose the filters and thresholds that best suit their application. For example, the required base quality could be lowered for highly degraded samples, or the minimum variant allele frequency (VAF) lowered for very deeply sequenced samples. These settings can easily be reported (or supplied as a configuration file) in published work that uses FiNGS, thus ensuring reproducible results without the need of custom scripts.

### Implementation

FiNGS is implemented in Python 3 and easy to install with a myriad of options for different computational environments. It is available via Bioconda [5], PyPI, directly from GitHub as source code, or as a Docker image which is also compatible with Singularity [6].

**Fig. 1:**
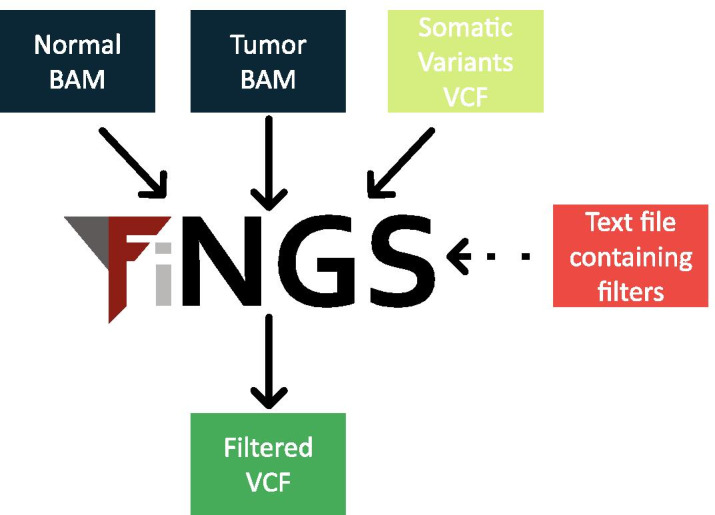
The FiNGS workflow. A user supplies the BAM files from a pair of matched normal and tumor samples, along with a standard VCF file listing somatic mutations and a text file detailing the filters and thresholds to be used. FiNGS outputs a filtered VCF file, along with a number of additional files that the user may find useful

The FiNGS workflow is shown in Fig. 1. The required inputs are a standard VCF file from any somatic variant caller along with the tumor and normal BAM files of sequencing data used to generate it. FiNGS is optimized for use with Illumina paired-end sequencing data. Although this represents the majority of sequencing data available, there are other sequencing platforms and users must choose filters and parameters appropriate to their own data, as there may be patterns of errors unique to them which we have not considered.

An optional additional input is a text configuration file specifying the filters and thresholds to use, although two default configuration files are supplied with the software, one with filters and thresholds we have selected and one that replicates the ICGC filtering recommendations. FiNGS operates a two-stage procedure. First, it calculates a wide range of metrics for every potential variant listed in the VCF using the Pysam package to access the BAM files and stores the metrics in gzipped compressed text files. These metrics are not otherwise available in the source VCF or BAM files. Second, these metrics are used to apply the filters specified by the user, resulting in a standard filtered VCF with headers detailing the filters and thresholds, along with a log file, a text file containing filter results for every variant and plots summarizing the pass and fail counts for each filter.

The metric-gathering process is the most expensive in terms of computation and time. However, we have taken the following steps to ameliorate this. First, we have used the joblib package to implement parallelization. Second, FiNGS will identify if previously calculated metrics are available to avoid recalculation in the event of changing filter thresholds. This makes it fast and simple for a user to filter a data set using multiple different filters and thresholds, while also offering protection against user-input error.

Default filters were designed based on features suggested by ICGC recommendations [4] and heuristics such as depth of coverage and variant allele frequency (VAF). All filters and the rationale for each are described in detail in the documentation with suggested thresholds (Additional file 1: Table 1). At runtime a tab-delimited text file specifying the desired filters and thresholds is supplied by the user and only these filters will be applied. FiNGS comes with a default set of filters and thresholds. However, the exact ICGC standards can be invoked using the “–ICGC" flag. Further, users can share their filtering methodologies simply by providing the tab-delimited configuration text file that lists filters and thresholds.

Training data was composed of three separate sets of samples and was used to optimize parameters for each filter, attempting to maximize both recall and precision. Two synthetic data sets from a previous study [7], representing a targeted panel sequenced to 520 × depth and whole exome sequencing (WES) sequenced to 70 × depth, both using Illumina reads with normal contamination ranging from 0 to 90%.

The third data set was whole genome sequencing (WGS) sourced from the Genome In A Bottle (GIAB) consortium, sequenced from a physical mixture of two extremely well-characterized human genomes. A mixture of 30% NA12878 (“tumor”) and 70% NA24385 (“normal”) was sequenced to 90 × depth, with a 30 × depth sample of 100% NA24385 acting a matched normal sample.

Validation was performed using the same tumor-normal pair of deeply sequenced whole genomes from a medulloblastoma patient used by the ICGC to produce their recommendations on variant filtering [4].

All data were aligned using BWA version 0.7.12 [8], followed by sorting and deduplication using Picard version 2.13 [9]. Training data was aligned to reference genome GRCh37 and validation data aligned to reference genome GRCh38. Variants were called using default parameters with both MuTect version 1.1.7 [10] and Strelka2 version 2.8.3 [11].

Software performance was tested by calculating the precision, recall and F_1_ score for each condition using detection of known true positive mutations. Recall is a measure of the proportion of true positives recovered after filtering, precision is a measure of the proportion of filtered results that are true positives and the F_1_ score is the harmonic mean of recall and precision; all range between 0 and 1, with values closer to 1 being better. Recall, precision and F_1_ score are defined as:

$$Recall = \frac{TP}{{TP + FN}}$$

$$Precision = \frac{TP}{{TP + FP}}$$

$$F_{1} = \frac{2TP}{{2TP + FP + FN}} = 2 \cdot \frac{precision \cdot recall}{{precision + recall}}$$

## Results

**Fig. 2:**
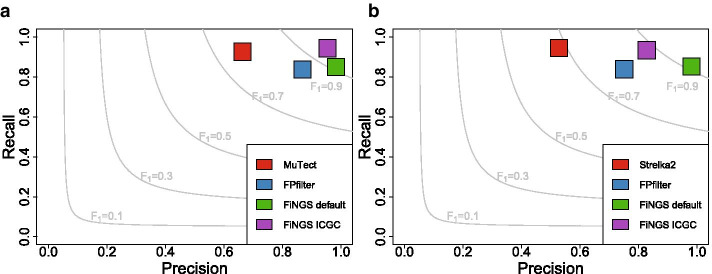
Recall versus precision plots for validation data sets. Filtering results for MuTect (**a**) and Strelka2 (**b**). Contour lines demarcating thresholds for F_1_ scores are marked and labelled in gray. FPfilter and FiNGS increased the precision of the results, for a small cost to recall. Overall, the default and ICGC modes of FiNGS produced the best results

The ICGC validation data contained 1262 true positive SNVs which were used to calculate the recall, precision and F_1_ scores for raw variant calls from MuTect and Strelka2, and after filtering by FPfilter and FiNGS using both the default settings and ICGC settings. Results are summarized in Fig. 2. With no further filtering, MuTect (Fig. 2A) and Strelka2 (Fig. 2B) had excellent recall (0.93 and 0.95), calling most of the true positives. However, they also had poor precision (0.66 and 0.53), clearly illustrating the need for post-call filtering, as nearly half the results were false positives, resulting in moderate F_1_ scores (0.77 and 0.68).

FPfilter was effective at increasing overall precision in MuTect (0.87) and Strelka2 (0.75), albeit with lower recall, resulting in improved F_1_ scores (0.85 and 0.79). FiNGS achieved superior results, regardless of the variant caller used, both with the default filters and ICGC filters. The FiNGS default filters performed similarly with both MuTect and Strelka2 calls, with a large increase in precision at the cost of a reduction in recall (recall, precision and F_1_ scores were 0.85, 0.98 and 0.91 in both). Results with the ICGC filters applied using FiNGS were more variable, giving the best observed results in MuTect and lower precision but higher recall than the default FiNGS filters with Strelka2. The default settings of FiNGS were developed using BWA-aligned data. Using other aligners may therefore require alternative settings when filtering based on parameters that are generated by aligners, such as alignment quality score. Despite the large number of filters available, we find that excluding variants with low VAF tends to have the most pronounced effect.

## Conclusions

These data demonstrate the value in further filtering variant calls beyond what is initially emitted by somatic variant callers. The type and stringency of filtering is a careful balance between the reduction of false positives (increased precision) and excluding true positives (decreased recall). We have developed FiNGS, which substantially increases the precision of results, providing high quality variants for further analysis. The filters and thresholds to be applied are fully configurable by the user, allowing simple, consistent, flexible and reproducible filtering of somatic variants, along with an easy method of sharing filtering strategies between users. We encourage users to think critically about their filtered results, including examining the plots produced so that they have confidence that they are not over or under-filtering.

## Availability and requirements

- Project name: FiNGS (Filters for Next Generation Sequencing)
- Project home page: https://github.com/cpwardell/FiNGS
- Operating system(s): Platform independent
- Programming language: Python 3
- Other requirements: e.g. Python 3.5 or higher
- License: Apache 2.0
- Any restrictions to use by non-academics: None

## Supplementary Information

**Additional file 1** Supplementary Table 1: Filters included in FiNGS, with descriptions and rationales for each of them. Also includes default values in FiNGS, ICGC recommendations and FPfilter values. Using a subset of the validation data, we calculated the recall, precision and F1 scores for each set of parameters in each filter and report the parameter with the highest F1 score.

## Data Availability

Source code and full documentation is available at GitHub https://github.com/cpwardell/FiNGS, along with links to downloads at PyPI, Bioconda and the Singularity-compatible Docker container. All data sets are available from public repositories as follows; Bohnert et al.’s synthetic data (https://doi.org/10.5281/zenodo.556347), the Genome In A Bottle (GIAB) consortium admixtures (https://jimb.stanford.edu/giab) and the ICGC medulloblastoma data is available at the European Genome-phenome Archive under accession number EGAS00001001539.

## Abbreviations

BAM: binary alignment map
FN: false negative
FP: false positive
GATK: genome analysis tool kit
ICGC: international cancer genome consortium
SNV: single nucleotide variant
TP: true positive
VCF: variant call format
